# Physician Associate and General Practitioner Consultations: A Comparative Observational Video Study

**DOI:** 10.1371/journal.pone.0160902

**Published:** 2016-08-25

**Authors:** Simon de Lusignan, Andrew P. McGovern, Mohammad Aumran Tahir, Simon Hassan, Simon Jones, Mary Halter, Louise Joly, Vari M. Drennan

**Affiliations:** 1 Department of Clinical and Experimental Medicine University of Surrey, Guildford, GU2 7XH, United Kingdom; 2 Division of Population Health Sciences and Education St. George’s University of London, London, SW17 0RE, United Kingdom; 3 AT Medics, St. Charles Hospital, Exmoor Street, London, W10 6DZ, United Kingdom; 4 Department of Population Health NYU School of Medicine, 227 East 30th Street, New York, New York, 10016, United States of America; 5 Faculty of Health, Social Care & Education, Kingston University & St. George’s University of London, London, United Kingdom; 6 Social Care Workforce Research Unit King’s College London, Strand, London, WC2R 2LS, United Kingdom; University of Sydney, AUSTRALIA

## Abstract

**Background:**

Physician associates, known internationally as physician assistants, are a mid-level practitioner, well established in the United States of America but new to the United Kingdom. A small number work in primary care under the supervision of general practitioners, where they most commonly see patients requesting same day appointments for new problems. As an adjunct to larger study, we investigated the quality of the patient consultation of physician associates in comparison to that of general practitioners.

**Method:**

We conducted a comparative observational study using video recordings of consultations by volunteer physician associates and general practitioners with consenting patients in single surgery sessions. Recordings were assessed by experienced general practitioners, blinded to the type of the consulting practitioner, using the Leicester Assessment Package. Assessors were asked to comment on the safety of the recorded consultations and to attempt to identify the type of practitioner. Ratings were compared across practitioner type, alongside the number of presenting complaints discussed in each consultation and the number of these which were acute, minor, or regarding a chronic condition.

**Results:**

We assessed 62 consultations (41 general practitioner and 21 physician associates) from five general practitioners and four physician associates. All consultations were assessed as safe; but general practitioners were rated higher than PAs in all elements of consultation. The general practitioners were more likely than physician associates to see people with multiple presenting complaints (p<0.0001) and with chronic disease related complaints (p = 0.008). Assessors correctly identified general practitioner consultations but not physician associates. The Leicester Assessment Package had limited inter-rater and intra-rater reliability.

**Conclusions:**

The physician associate consultations were with a less complex patient group. They were judged as competent and safe, although general practitioner consultations, unsurprisingly, were rated as more competent. Physician associates offer a complementary addition to the medical workforce in general practice.

## Introduction

Physician associates (PAs), known internationally as physician assistants, are a type of mid-level practitioner new to the United Kingdom (UK). The PA is “a new healthcare professional who, while not a doctor, works to the medical model, with the attitudes, skills and knowledge base to deliver holistic care and treatment within the general medical and/or general practice team under defined levels of supervision” by a physician [1]. PAs developed in the United States of America (USA) in the mid-1960s to tackle a national shortage of doctors and to increase access to healthcare [2,3]. In recent years, the profession has begun to spread globally including to the Netherlands, India, Canada and Australia [4].

The first UK-trained PAs graduated in 2009. The PA training programme in the UK is a two year full-time course for graduates, generally with life sciences degrees, and is delivered by an increasing number of higher education institutions, supported by the national health care workforce plans [5]. After graduation PAs are encouraged to register on the PA Managed Voluntary Register (PAMVR), held by the Faculty of Physician Associates at the Royal College of Physicians [6]. It has been suggested that there is a need to develop stronger governance frameworks for the profession [7] and the Faculty has been working for PAs to be included in the state regulatory processes for health professions. One consequence of their exclusion from the regulatory process is that PAs in the UK cannot currently prescribe medications.

Currently, the majority of the PAs’ workload in UK primary care is in providing same day and urgent consultation appointments [8]. This is similar to their role in primary care in the USA and elsewhere [9]. A systematic review [9] highlighted the lack of evidence as to the contribution PAs made to general practice (known as family physician services outside the UK). As an adjunct to a large mixed methods study [10], we investigated the quality of the patient consultation, by PAs compared with general practitioners (GPs) as judged by independent experienced GPs from video recordings.

Video analysis of the consultation is an established part of general practitioner training in the UK, providing insight into the content and complexity of the clinical consultation [11]. Advanced techniques have been used to more precisely observe the interaction between clinician, patient and computer [12] and this approach was used here.

## Methods

This was a comparative, observational study of video recorded PA and GP primary care consultations.

### The sample

PAs and GPs were recruited from the 12 practices participating in a larger study of the role of PAs (six practices with PAs and six without in the East, South West and South East of England). Details of the parent study and practice recruitment is given elsewhere [10]. PAs and GPs, in the study practices, were invited to participate in the video observation study and written informed consent was gained from each practitioner prior to recording. The volunteer GPs and PAs each identified one specific clinical session in which they saw same day and urgent patient appointments for the video observations to be made. These sessions were in addition to those reported from the parent study [13].

Adult patients attending for same-day appointments in these sessions were invited to participate in the study by the researchers [MH, LJ, WC, JY]. Patients were informed about the study and video recording prior to their consultation, as they arrived for their appointment, and written consent was obtained from all participating patients by the researchers. Patients were informed about their right to withdraw consent for video recording at any stage. Recording started as the patient entered the room and ceased when they left the room. On leaving the consultation, the researchers asked patients whether they continued to consent to the recording being used or if they wished it to be deleted. Practitioners also consented for inclusion of each recording at the end of the clinical session. There was no data collected from the electronic patient record or other sources outside the consultation.

### The data

Consultations were recorded in 2012, using an adapted version of the Activity Log Files Aggregation (ALFA) toolkit which is a recording technique designed to facilitate precise observation of the consultation [14].

Recording was restricted to two cameras per consultation, one with a wide-angle view of the consultation and the other focused on the practitioner; this was to comply with the requirements of the NHS research ethics committee. Cameras were positioned so as to not record intimate examinations. The two video feeds were combined to produce a dual-channel video used for assessment. The resulting videos were stored on encrypted and password protected hard drives. Each consultation was stored in full and in an edited version, which removed any evidence which revealed the role of the consulting clinician. These included statements made by the clinician e.g. “I need to get this prescription signed by the doctor”, periods where PAs left the room to get prescriptions signed, and references made by patients e.g. call the clinician “doctor”.

### Analysis

The analysis comprised of assessment of the quality of the consultation, whether experienced GPs could correctly identify GPs or PAs from the videos and comments on the safety of the consultations, and an analysis of patient case mix. Each of these components is described below.

#### Assessment of the quality of the consultation

Consultation quality was assessed by a group of four GP assessors using the Leicester Assessment Package. The Leicester Assessment Package has been demonstrated to have validity and reliability across levels of clinical competence [15–17]. However, the reliability of the package has been questioned by others [18, 19] although no better consultation assessment tool has yet been universally accepted. It has been used for consultation assessment internationally [20], with practice nurses i.e. nurses working in family physician offices [21] and medical students [22].

Assessment of consultation quality is divided into the key areas shown in Table 1.

**Table 1:** Areas of assessment of the clinical consultation as described by the Leicester Assessment Package. Record keeping was not assessed in our study therefore an adjusted weighting was used to calculate a global score.

Area of assessment	Weighting (%)	Adjusted weighting (%)
Interview/history taking	20	22.2
Physical examination	10	11.1
Patient management	20	22.2
Problem solving	20	22.2
Behaviour/relationship with patients	10	11.1
Anticipatory care	10	11.1
Record keeping	10	Not assessed

Each component was graded A to E using the Leicester Assessment Package scoring criteria:

**A:** Demonstrates mastery of all (or almost all) components consistently and to the highest standard.
**B:** Demonstrates mastery of all (or almost all) components consistently and to a high standard, and some to the highest standard.
**C+:** Consistently demonstrates capability in all (or almost all) components to a satisfactory standard—some to a high standard.
**C:** Demonstrates capability in all (or almost all) components to a satisfactory standard but tends to lack discrimination, organisation and good time management.
**D:** Demonstrates inadequacies in at least one component. Lacks discrimination and/or organisation. Tends to perform inconsistently.
**E:** Demonstrates major omissions and/or serious defects. Grossly unacceptable standard overall.

Assessors were experienced GPs from teaching and training primary care practices. Assessing clinicians were required to attend two training sessions (led by SdeL) to familiarise them with the video format and to provide training in use of the Leicester Assessment Package. The two consultation videos used for training purposes were excluded from the final assessment. Each consultation was assessed by a minimum of two clinicians independently. Assessors were not able to comment on record keeping as there was no third video channel (the clinician’s computer screen) incorporated as part of the ALFA recording. This section of the Leicester Assessment Package was therefore excluded from the analysis with global scores adjusted accordingly.

Assessors were blinded to the role of the clinician performing each consultation. Assessors were required to state whether they thought the consulting clinician was a GP or a PA based on the consultations they had viewed by that clinician.

Following assessment of all the videos by the GP assessors, a workshop was undertaken with all the assessors which was recorded, transcribed and analysed. As part of this GP assessors were asked to comment on whether they felt the practitioners they had viewed were safe to practice and for their comments on the use of the Leicester Assessment Package as a method of assessment.

A Mann-Whitney U-test was used to compare differences between PAs and GPs in median scores, for each area of assessment of the Leicester Assessment Package, to identify statistical significance (p>0.05). A global score was calculated from the area of assessment scores using the adjusted weightings in Table 1. Each consultation was assessed by two assessors and these data compared to interrogate inter-rater reliability. Inter-rater reliability was calculated for each area of assessment of the Leicester Assessment Package using quadratic weighted kappa and Spearman’s rank correlation. The assessment process consisted of two rounds of assessment. As part of the second round ten videos were reviewed by the same pair of assessors as in the first round. These data were used to analyse intra-rater reliability (test-retest reliability) assessed using Cronbach’s alpha. Statistical analyses were performed using the software package R.

#### Case mix analysis

In order to identify case mix differences between PA and GP consultations each consultation recording was reviewed by a clinician [AMcG] to determine the number of presenting complaints, the category of the presenting complaint (acute, chronic, or minor/symptoms), and the number of relevant chronic conditions.

The number of presenting complaints was defined as the number of unrelated primary complaints raised by the patient during each consultation. The category of the presenting complaint was based on the system devised by de Jong et al [23] and extended by Halter et al [24]. Each presenting complaint was categorised as follows: acute conditions were only those included in the list of acute conditions defined by de Jong et al. [23], chronic conditions were those on the list of chronic conditions defined in the Quality Outcomes Framework or another condition which has lasted for over a year as described by the patient, and minor/symptoms as conditions on the list of minor/symptoms conditions defined by de Jong et al. [23] or another condition which had lasted for less than a year and not classified as an acute condition. Relevant chronic conditions were defined as long-term conditions discussed in the consultation and relevant or related to one or more of the presenting complaints (but not a primary reason for consultation).

The relationship between the patient’s consultation with a PA or GP and the number of presenting complaints, nature of presenting complaint, and number of relevant chronic conditions was explored using a Chi-squared analysis.

### Ethical considerations

Research ethics approval was granted by NHS Research Ethics Committee (REC) South East Coast–Surrey (REC reference number: 10/H1109/28).

## Results

Consultations were recorded from five GPs and four PAs. Two GPs were female and two PAs female. The average number of years of experience since qualification was 19.2 (±8.9) in GPs and 7.0 (±6.1) in PAs. 86 patients were approached; 73 consented to the video; one subsequently withdrew consent for their consultation to be included following the consultation; no videos were withdrawn by clinicians following filming. A total of 62 patient consultations were used in the final assessment (the remaining videos were randomly selected for use in the training sessions); these comprised 41 GP and 21 PA consultations. One patient requested that only a single camera was used during their consultation. This consultation was included in the analysis with only a single video feed, the recording on the second camera was deleted.

### Quality of Consultation

**Fig 1:**
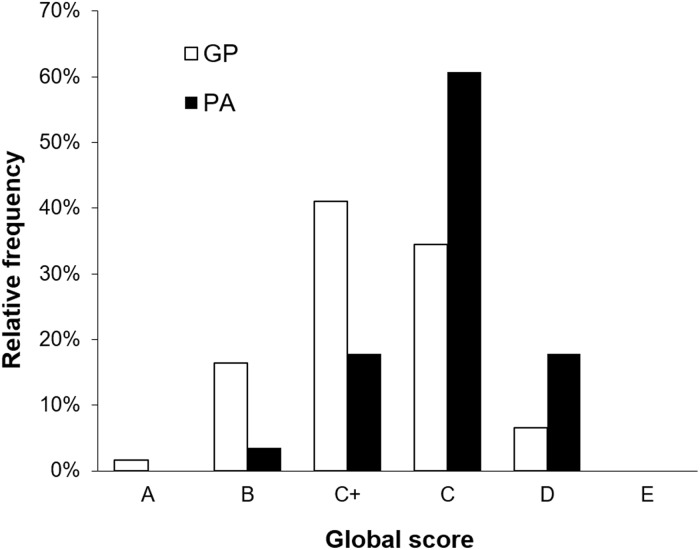
The relative frequency of global consultation scores from 124 assessments of 62 consultations; 41 general practitioner (GP) and 21 physician assistant (PA).

The average global score was C. The individual clinician averaged global scores were GP; C, C+, C+, C+, C+ and PA; C, C, C+, C+. Global scores for individual consultations ranged from D to A (Fig 1) however no PAs were given an overall grade A for any consultation. All consultations were above the minimum standard with no consultation given grade E.

Median scores were higher for GP consultations than PA consultations in each domain of the Leicester Assessment Package (Table 2).

**Table 2:** Collated scores for 41 general practitioner (GP) consultations and 21 physician assistant (PA) consultations evaluated using the Leicester Assessment Package. CI = confidence interval, IQR = Interquartile range.

	Median scores (IQR)		Mann-Whitney U test p value
Area of assessment	PAs	GPs	
Interview/history taking	C (D to C+)	C+ (D to B)	0.011
Physical examination	C (D to C)	C+ (C to B)	0.003
Patient management	D (D to C)	C (D to C+)	< 0.001
Problem solving	C (E/D to C+)	C+ (C to B)	< 0.001
Behaviour/relationship with patients	C (D to B)	C+ (D to B)	0.009
Anticipatory care	D (D to C)	C (D to C)	0.024
Record keeping	Not assessed	Not assessed	-
**Global score**	**C (D/C to C+)**	**C+ (C to C+)**	**0.022**

Inter-rater reliability of the Leicester Assessment Package was limited (Table 3). In weighted kappa analysis scores for behaviour/relationship with patient demonstrated substantial agreement; interview/history taking and global score, moderate agreement; physical examination, patient management, and problem solving, fair agreement; and anticipatory care no agreement. Spearman’s rank analysis produced comparable results: strong positive correlation between scores for behaviour/relationship with patient, interview/history taking, and global score; weak correlation, for physical examination, patient management, and problem solving; and no correlation, for anticipatory care.

**Table 3:** Weighted kappa and Spearman’s rank analyses of inter-rater reliability of consultation scores for 41 general practitioner (GP) consultations and 21 physician assistant (PA) consultations evaluated using the Leicester Assessment Package.

	Quadratic weighted kappa		Spearman's rank	
Area of assessment	kappa	95% CI	Correlation co-efficient	p value
Interview/history taking	0.550	0.363–0.737	0.585	<0.001
Physical examination	0.294	0.083–0.504	0.315	0.009
Patient management	0.330	0.142–0.518	0.433	<0.001
Problem solving	0.246	0.001–0.484	0.258	0.024
Behaviour/relationship with patients	0.710	0.601–0.819	0.727	<0.001
Anticipatory care	0.124	-0.287–0.536	-0.149	0.148
**Global score**	**0.602**	**0.428–0.777**	**0.650**	**<0.001**

CI = confidence interval.

Ten consultations were assessed twice by the same pair of raters giving a total of 20 repeat assessment scores. Intra-rater (rater-rater) reliability, assessed using Cronbach’s alpha, was good for interview/history taking, physical examination, problem solving, behaviour/relationship with patients, and global scores (Table 4). Poor intra-rater reliability was found for patient management. No intra-rater reliability was found for anticipatory care.

**Table 4:** Cronbach’s alpha analysis of intra-rater reliability of 20 pairs of consultation scores evaluated using the Leicester Assessment Package.

Area of assessment	Cronbach’s alpha	95% CI	P value
Interview/history taking	0.819	0.658–0.904	<0.001
Physical examination	0.764	0.554–0.875	<0.001
Patient management	0.488	0.032–0.729	0.020
Problem solving	0.758	0.543–0.872	<0.001
Behaviour/relationship with patients	0.838	0.693–0.914	<0.001
Anticipatory care	0.270	-0.380–0.614	0.165
**Global score**	**0.869**	**0.752–0.930**	**<0.001**

The assessors were also asked for comments on the Leicester Assessment Package. Assessors highlighted that there was no complexity domain in the assessment and agreed they would like to any future assessment tools to include a measure of consultation complexity.

All four raters attempted to identify whether each clinician was a GP or PA based on the consultations they had observed. GPs were correctly identified 90% (95% CI 75–100; p = <0.001) of the time. PAs were correctly identified 56% (95% CI 31–81; p = 0.227) of the time.

During the workshop with GP assessors they stated that all consultations and practitioners appeared to be practicing safely.

### Case mix analysis

From the 62 consultations recorded there were a total of 85 presenting complaints (mean 1.4 per consultation; range 1 to 3 per consultation). Of the 41 GP consultations 18 patients (44%) had two distinct presenting complaints and three (7%) had three distinct presenting complaints. Of the 21 PA consultations one patient (5%) had two presenting complaints and none had three. These differences were statistically significant (χ^2^ = 14.2, df = 2, p = 0.01).

Of the 85 presenting complaints one (1%) was related to an acute condition, 38 (45%) to chronic conditions, and 46 (54%) to minor/symptom conditions. Patients with presenting complaints relating to chronic conditions were more likely to consult a GP than a PA (χ^2^ = 14.2, df = 2, p = 0.01).

Fewer than half of the patients observed (28 of 62; 45%) had one or more chronic conditions relevant to their presenting complaint (but not the main reason for presentation); 8 of 21 (38%) of those consulting a PA and 20 of 41 (49%) of those consulting a GP. There was no significant relationship identified between having one or more chronic conditions related to the presenting complaint and the patient consulting a PA or GP (χ^2^ = 0.95, df = 1, p = 0.33)

## Discussion

### Principal findings

GPs were assessed to have performed better in all domains of the consultation than the PAs. All clinicians were judged as practising safely. However, the Leicester Assessment Package had limited inter-rater and intra-rater reliability. GP assessors were able to correctly identify GP consultations but failed to correctly identify 2 of 4 of the PAs. PAs in this study saw patients who largely attended with a single presenting complaint, whereas those who attended a GP had two or more presenting complaints. Patients seeing a GP were more likely to have one or more chronic diseases.

### Comparison with the literature

To our knowledge, there are no other published studies comparing competence in the consultation between GPs and PAs from video-recorded consultations. We identified only one study in which transcribed audio-recordings were analysed to compare the consultations of GPs and nurse practitioners, another type of mid-level practitioner who substitute for GPs in primary care [25]. It reported on a comparison of utterances rather than an assessment of all the consultation elements. Other comparative studies of nurse practitioners in primary care have analysed data only from patient records and questionnaires [26–30].

Patients consulting GPs had more presenting complaints and chronic diseases than those consulting the PAs. This supports evidence reported from England [13] and the USA [31]. The evidence of safety in consultation and also difficulty in determining the difference in the consulting style of some PAs from a GP is supported in our larger study using data from electronic patient records and patient report surveys [13]. PAs were found to be acceptable, effective and efficient in complementing the work of GPs and to generate better records of the consultation than GPs [13]. The GPs were rated more highly in the competency of their consultation which has not been reported before in comparison with PAs or nurse practitioners [31].

### Limitations of the method

Our study, through necessity, was small; capturing data from a total of nine clinicians. This limits the generalisability of the results.

In our clinician sample the majority of the GPs had substantially more years training and experience than PAs. Therefore in direct comparison you might expect them to perform better than PAs. In addition we identified differing patient case mix between the two types of practitioner which may limit the comparison of consultation performance.

The impact of video recording may have affected clinician behaviour differentially between PAs and GPs. Some of the GPs in the sample had previously had consultations recorded during their training or practice whereas none of the PAs had experience. One GP scheduled extra breaks into her clinic to prevent delays caused by the videoing process.

We were unable to employ the full ALFA toolkit as ethical restrictions prevented us from capturing a third view; the computer screen. This may have produced some inaccuracies in measuring the clinician-computer interaction time compared with previous studies; however this discrepancy is likely to be small. This prevented us from accurately measuring the record keeping component of the Leicester Assessment Package.

The inter-rater reliability for a number of sections of the Leicester Assessment Package was only fair, namely; physical examination, patient management, and problem solving. There was no inter-rater correlation between scores of anticipatory care. Therefore, the difference between PAs and GPs observed for these domains of the Leicester Assessment Package should be treated with extreme caution. However, good to excellent agreement was observed for behaviour/relationship with patient, interview/history taking, and global scores and we therefore conclude that the differences we observed in these domains are likely to represent genuine quality differences.

Previous concerns have been raised about the reliability of the Leicester Assessment Package 18,19 and the initial analysis of the package demonstrated some scoring inconsistencies [15]. Our findings support these concerns. We suggest that in depth analysis of the Leicester Assessment Package is required to establish which components have a good inter and intra-rater reliability and which components require adaptation.

We cannot exclude rater bias towards GP consultation styles as all the raters were GPs. They may therefore maybe predisposed to rate more highly a consultation style more similar to their own. Additionally blinding of GP raters to clinician type appears to have been incomplete with some clinicians identified correctly by all raters. However despite this incomplete blinding GP raters were not able to correctly identify all clinicians as PAs or GPs.

The ability of GP raters to comment on safe practice of clinicians was limited. Raters were not able to view the consultation notes recorded by practitioner or the prescriptions issued (although in most consultations the prescribed medications were described to the patient by the practitioner).

### Implications of the findings

It was interesting that experienced GPs could not differentiate PAs from GPs, and importantly they considered that PAs provide safe consultations. Bearing in mind that a graduate can be trained to be a PA in two years, compared with it taking nine years for a graduate going into medicine to become a GP, it is a credit to their training that they practice safely. However GPs performed consistently better across all domains of assessment than PAs. These differences may, in part, be accounted for by the greater duration of experience of the GPs included in this study, differing case mix, and perhaps partly by rater bias. Additional analysis with adjustment for these potential sources of error is required to see if this difference persists when comparing practitioners with a similar duration of clinical experience.

PAs’ role in the workforce is complimentary to that of GPs. They effectively treated cases that predominantly comprised patients with single presenting problems and were less likely to have a chronic disease. We did not collect any data to know whether this was due to patient or practice staff selection, or a combination of both. However, this suggests how PAs might be complimentary to the primary care workforce.

## Conclusions

PAs in the primary care work force, as well as GPs, were regarded as safe. They saw less complex cases. The average competence of PA consultations was rated as lower than those of GPs. There is a place for PAs in the primary health care team, probably best in a group practice setting where they can meet a wider range of patient needs.

## Ethical considerations

The research ethical review was undertaken by NHS Research Ethics Committee (REC) South East Coast–Surrey (REC reference number: 10/H1109/28).
